# The Use of Antidepressants, Anxiolytics, and Hypnotics in People with Type 2 Diabetes and Patterns Associated with Use: The Hoorn Diabetes Care System Cohort

**DOI:** 10.1155/2017/5134602

**Published:** 2017-01-23

**Authors:** R. Mast, S. P. Rauh, L. Groeneveld, A. D. Koopman, J. W. J. Beulens, A. P. D. Jansen, M. Bremmer, A. A. W. A. van der Heijden, P. J. Elders, J. M. Dekker, G. Nijpels, J. G. Hugtenburg, F. Rutters

**Affiliations:** ^1^EMGO+ Institute for Health and Care Research, VU Medical Center, Amsterdam, Netherlands; ^2^Department of Clinical Pharmacology and Pharmacy, VU Medical Center, Amsterdam, Netherlands; ^3^Department of Epidemiology and Biostatistics, VU Medical Center, Amsterdam, Netherlands; ^4^Julius Center for Health Sciences and Primary Care, University Medical Center Utrecht, Utrecht, Netherlands; ^5^Department of General Practice and Elderly Care Medicine, VU Medical Center, Amsterdam, Netherlands; ^6^Department of Psychiatry, VU Medical Center, Amsterdam, Netherlands

## Abstract

*Objective*. With depression being present in approximately 20% of people with type 2 diabetes mellitus (T2DM), we expect equally frequent prescription of antidepressants, anxiolytics, and hypnotics. Nevertheless, prescription data in people with T2DM is missing and the effect of depression on glycaemic control is contradictory. The aim of this study was to assess the prevalence of antidepressants, anxiolytics, and/or hypnotics use in a large, managed, primary care system cohort of people with T2DM and to determine the sociodemographic characteristics, comorbidities, T2DM medication, and metabolic control associated with its use.* Method*. The prevalence of antidepressants, anxiolytics, and/or hypnotics use in the years 2007–2012 was assessed in the Hoorn Diabetes Care System Cohort from the Netherlands.* Results*. From the 7016 people with T2DM, 500 people (7.1%) used antidepressants only, 456 people (6.5%) used anxiolytics and/or hypnotics only, and 254 people (3.6%) used a combination.* Conclusion*. We conclude that in our managed, primary care system 17% of all people with T2DM used antidepressants, anxiolytics, and/or hypnotics. Users of antidepressants, anxiolytics, and/or hypnotics were more often female, non-Caucasian, lower educated, and more often treated with insulin.

## 1. Introduction

In our current society, depression is present in approximately 20% of the people with type 2 diabetes mellitus (T2DM), twice as many when compared to the nondiabetic population [1]. Two recent meta-analyses have shown that depression increases the risk of developing T2DM and vice versa [2, 3]. Similar reciprocal associations have been observed for anxiety, sleep disorders, and T2DM [4, 5].

Depression, anxiety, and/or sleep problems are diagnosed using the Diagnostic and Statistical Manual of Mental Disorders IV (DSM-IV) [6] and are treated with psychotherapy or pharmacotherapy when the patient is unresponsive to the former [7]. First choice treatment is psychotherapy, while pharmacotherapy may possibly lead to poorer glycaemic control. However, studies on the effect of depression/pharmacotherapy on glycaemic control have been contradictory [8–10]. Despite the cautious treatment regimes, in Western society, antidepressants, anxiolytics, and hypnotics are by far the most often prescribed drugs [10, 11]: in the general population 6% are prescribed antidepressants and 7.5–9.9% are prescribed anxiolytics and/or hypnotics [8, 12].

While the prevalence of depression, anxiety, and/or sleep disorders in people with T2DM is high (20%) [13, 14], we expect equally frequent prescription of antidepressants, anxiolytics, and hypnotics. One study from Finland reported that antidepressant use among men and women who develop T2DM was 2 times higher than the use in nondiabetic individuals [15]. Nevertheless, to our knowledge prevalence data of antidepressants, anxiolytics, and hypnotics use in people with T2DM are scarce. This may be due to the fact that people with T2DM are often excluded from trials on depression treatment [16]. In addition, all previous studies [5–8, 11–13] on the coincidence with T2DM assessed depression, anxiety, and/or sleep disorders by interview or questionnaire, with no information on pharmacological treatment. Our first aim is therefore to assess the prevalence of antidepressants, anxiolytics, and hypnotics use in a population-based cohort of people with T2DM.

Patterns of antidepressants, anxiolytics, and/or hypnotics use are well known in the general population [11, 17]. Antidepressants, anxiolytics, and/or hypnotics are more often prescribed to women, compared to men [11]. Furthermore, lower education level, older age, and non-Caucasian ethnicity have been associated with use of antidepressants, anxiolytics, and/or hypnotics [11]. While people with T2DM often have a lower level of education and higher age, we expect higher levels of antidepressants, anxiolytics, and/or hypnotics use in people with T2DM compared with the general population [17, 18]. Up until now the patterns of use of antidepressants, anxiolytics, and/or hypnotics in people with T2DM have not been investigated.

However, identifying and treating depression, anxiety, and sleep problems has become an increasingly important component of the diabetes management. Nevertheless, we do not know if people with T2DM are more often treated with antidepressants, anxiolytics, and/or hypnotics and information on the determinants of use.

Therefore, the overall aim of this study was to assess the prevalence of antidepressants, anxiolytics, and/or hypnotics use in a large, managed, primary care system cohort of people with T2DM and to determine the sociodemographic characteristics, comorbidities, T2DM medication, and metabolic control associated with its use.

## 2. Methods and Materials

### 2.1. Data Source

#### 2.1.1. Hoorn Diabetes Care System Cohort

Data were derived from the Hoorn Diabetes Care System (DCS) Cohort, a prospective using clinical care data, which is described in detail elsewhere [19]. In short, the DCS Cohort uses a centrally organized managed care plan for treatment of people with T2DM with contracted health care providers. The organization of the DCS is therefore responsible for all the quality of diabetes care in the region of West-Friesland in the Netherlands, a region with about 200,000 inhabitants. The DCS Cohort encompasses the care provided by a patient's primary care physician and secondary care, according to the Dutch treatment guidelines for T2DM [20]. The data from the annual assessment of the people with T2DM, which is organized centrally by the DCS, was used in this study.

Starting from 1st of October 1996, all the people from the catchment region who have been diagnosed with T2DM enter our prospective dynamic DCS Cohort, which currently holds 9118 people with T2DM with at least one measurement at the 31st of December 2012. For each patient, the year of entry to the DCS Cohort was considered to be the baseline measurement and the reported annual data will therefore contain data from people with incident T2DM and prevalent T2DM. We estimated the point prevalence of the use of antidepressants, anxiolytics, and hypnotics over each year and showed in more detail the participant characteristics in the year 2011, as that year the digital registration was implemented and thus the dataset most complete. For our current study, we had medication use data of all patients included and we excluded only patients who did not visit the DCS in the year 2011 (*n* = 2102), which resulted in a cohort of 7016 people with T2DM. The DCS Cohort has anonymous computer records and people with T2DM were informed on the use of these records for research purposes. The characteristics of the Hoorn Diabetes Care System Cohort in the year 2011 are described in Table 1. The Medical Ethical Committee of the VU University Medical Center has approved this study.

#### 2.1.2. The New Hoorn Study Cohort, General Population

The New Hoorn Study is a population-based cohort, which is representative for the general Dutch population. In short, in 2006-2007, a population-based survey was carried out in the Dutch city of Hoorn, a city in the middle of the West-Friesland region. The eligible population consisted of 6180 men and women, aged 40–75 years, that were randomly selected from the municipal registry. Of the eligible participants, 45% agreed to participate, resulting in a population-based cohort of 2807 participants. As this population-based cohort is sampled from the general population within the catchment area of the Diabetes Care System Cohorts, is about the same age (max 10 years difference), adheres to the same level of care, and shares a study location, nurses, and study protocols, we believe it is the optimal comparison cohort. The characteristics of the population-based cohort in the year 2007 are described in Supplement Table  1 in Supplementary Material available online at https://doi.org/10.1155/2017/5134602. In short, this population-based cohort was aged 53.4 ± 6.7 years; similarly to the T2DM cohort about 50% was male, and, however, as it is a population-based cohort only 7.1% had prevalent diabetes according to the WHO criteria.

### 2.2. Measurements

At baseline and each year following, when the patients visit the Diabetes Care System, the following measures are taken.

#### 2.2.1. Medication

Information on current medication use was registered yearly at the annual visit by checking the dispensing labels of the medication brought in by people with T2DM.

1. Antidepressants, anxiolytics, and hypnotics: we categorized antidepressants, anxiolytics, and hypnotics based on their ATC codes, namely, N06A for antidepressants, N05B for anxiolytics, and N05C for hypnotics and sedatives. Since the division between anxiolytics and hypnotics depends on the indication, which we did not have, we combined anxiolytics and hypnotics use into one group.
2. People using diazepam enemas and/or hydroxyzine were not categorized as anxiolytic users, because diazepam enemas are more often prescribed for the emergency management of epileptic seizures and hydroxyzine is often prescribed against allergies. Diazepam enemas and hydroxyzine uses were therefore not included in this analysis.
3. People with T2DM were divided into 4 groups: (1) no use of antidepressants, anxiolytics, and/or hypnotics (people who are not taking the medications of interest at the time of the annual consultation); (2) only use of antidepressants; (3) only use of anxiolytics and/or hypnotics; (4) combined use of antidepressants and anxiolytics and/or hypnotics.
4. T2DM medication: we defined three groups, based on ATC codes: diet only (no medication), insulin analogues (A10A), and oral glucose lowering agents (OGA, A10B). The OGA group contains the following OGAs: metformin, sulphonylurea, thiazolidinediones, alfa glucosidase inhibitors, DPP4 inhibitors, meglitinides, and GLP-1 receptor agonists. Most patients using insulin analogues were using as well OGA.

#### 2.2.2. Sociodemographic Patterns

During the annual visit to the research center, several sociodemographic factors were assessed in an interview, performed by the research nurse. Age and gender were self-reported. Education level was assessed using the question: “What is your highest completed educational level?” The response alternatives were 1: primary education, 2: secondary education, practical training, 3: prevocational secondary education, 4: vocational training, 5: general secondary education or preuniversity education, 6: professional university education, and 7: university. Educational level was divided into 3 groups: low (levels 1-2), middle (levels 3–5), and high (levels 6-7). Ethnicity was self-reported based on the questions: “Indicate country of birth of your biological father and mother.”

#### 2.2.3. Comorbidities

Information on history of cardiovascular disease, including myocardial infarction, transient ischaemic attack, and stroke was self-reported.

#### 2.2.4. HbA1c Levels

HbA1c levels (%) were assessed from a fasted blood sample, using DCCT standardized reversed-phase cation exchange chromatography (HA 8160 analyzer, Menarini, Florence, Italy). HbA1c levels were detected using dual-wavelength colorimetric method (415–500). The intra-assay coefficient of variation was 0.6% at a mean level of 4.9% and the interassay CV was 0.8% at a mean level of 5.5%.

#### 2.2.5. Weight and Height

Weight (kg) and height (cm) were measured annually during the visit in the research center, while barefoot and wearing light clothing. BMI (kg/m^2^) was calculated by weight in kilograms divided by the square of height in meters.

### 2.3. Statistical Analysis

The patients characteristics of users and nonusers were reported as mean and standard deviation for continuous variables and, in the case of a skewed distribution, the medium with 25th and 75th percentile (diabetes duration).

We compared patterns of users and nonusers, using descriptive summary statistics, including One-Way ANOVA for mean levels, with Chi-square tests (linear-by-linear association) for proportions and Kruskal-Wallis test for median levels.

Subsequently, a model was created using multivariate logistic backward regression analysis. In this analysis we assessed which available patient and disease determinants at baseline were associated with antidepressants, anxiolytics, and/or hypnotics use. All determinants were entered simultaneously using backward elimination method, leading to a model including only significant (*p* < 0.5) determinants. Finally, we reported data on the prevalence of antidepressants, anxiolytics, and/or hypnotics use in the general population.

Statistical analyses were performed with SPSS version 20.0 (SPSS Inc., Chicago, IL) and a two-sided *p* value below 0.05 was considered statistically significant.

## 3. Results

### 3.1. Population

The population characteristics of the Hoorn Diabetes Care System Cohort of people with T2DM in the year 2011 are described in Table 1 and for the years 2007–2012 in Supplementary Table 2. In 2011, the cohort consisted of an equal amount of women and men with T2DM, with a mean age of 66.3 years (SD 11.7) and 22.5% of non-Caucasian ethnicity, while 40% had a low level of education. Most people with T2DM in the cohort used OGAs (60.1%) or insulin (combined with OGA (22.1%)) and the majority was overweight (30.2 kg/m^2^, SD 5.4), but well controlled as shown by a mean HbA1c level of 52 mmol/mol (6.9%).

### 3.2. Antidepressants and Anxiolytics and/or Hypnotics Use

**Figure 1:**
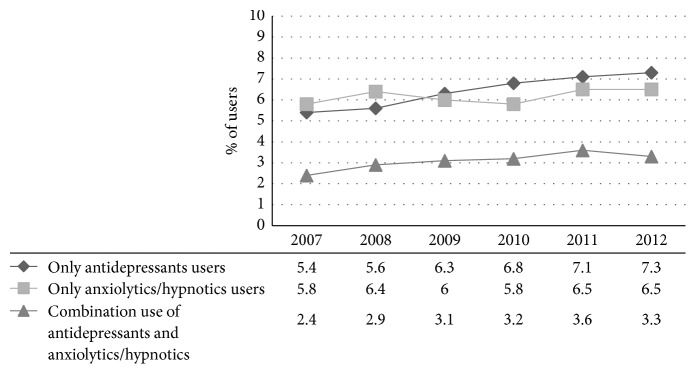
Prevalent use of antidepressants, anxiolytics, and/or hypnotics for the years 2007–2012 in the Hoorn Diabetes Care System Cohort.

In our cohort of people with T2DM, the prevalence of antidepressants, anxiolytics, and/or hypnotics use was 17.2%: with 7.1% using an antidepressant only, 6.5% using anxiolytic and/or hypnotic only, and 3.6% using a combination of antidepressants and anxiolytics and/or hypnotics.  Figure 1 showed the prevalence of antidepressants, anxiolytics, and/or hypnotics use between 2007 and 2012. In the total population, the prevalence of antidepressants use was 5.4% in 2007 and increased to 7.3% in 2012. Also, the prevalence of anxiolytics and/or hypnotics use increased, starting at 5.8% and increasing to 6.5% in 2012. With regard to patients using a combination of antidepressants and anxiolytics and/or hypnotics, the prevalence of use increased from 2.4% in 2007 to 3.3% in 2012.

### 3.3. Sociodemographic Patterns

Table 2 depicts the sociodemographic patterns stratified for antidepressant, anxiolytics, and/or hypnotics users versus nonusers in the year 2011. Compared to nonusers, users were more often female: 44.3% of the nonusers were female, while 61.8%–74.8% of users were female (*p* < 0.001). We also observed differences in ethnicity with 21.2% of the nonusers being non-Caucasian versus 26.2%–29.8% of the users (*p* < 0.001) as well as education level, with 42.7% of nonusers having a lower education level versus 48.0%–52.8% of users (*p* < 0.001). Further, compared to nonusers, those on anxiolytics and/or hypnotics (*p* < 0.001) as well as users of antidepressants and anxiolytics/hypnotics (*p* < 0.02) were significantly older. No significant age differences were observed in the users of antidepressants only.

### 3.4. Comorbidities

Nonusers had less comorbidities (myocardial infarction, transient ischaemic attack, and stroke), compared to people with T2DM on anxiolytics and/or hypnotics (15.4% versus 26.8%, *p* < 0.001). No differences in comorbidities were observed in nonusers versus antidepressant or anxiolytics and/or hypnotics users.

### 3.5. T2DM Treatment and Glycaemic Control

Finally, compared to nonusers, those people using antidepressants more often used insulin as T2DM treatment (21.5% versus 27.2%,  *p* < 0.001). No differences were observed in T2DM treatment between nonusers and anxiolytics and/or hypnotics users. Additionally, no significant differences were observed in continuous HbA1c or cut-off variables for HbA1c levels (≥53 mmol/mol) between users and nonusers. With regard to BMI, compared to nonusers, users of antidepressants had significant higher BMI levels compared to nonusers (31.2 kg/m^2^ versus 30.1 kg/m^2^, *p* < 0.001).

### 3.6. Antidepressants, Anxiolytics, and/or Hypnotics Users

Antidepressants, anxiolytics, and hypnotics users had a statistically significantly higher likelihood of being women and non-Caucasian and have more often comorbidities, compared to nonusers of antidepressants, anxiolytics, and hypnotics (Table 3).

### 3.7. Antidepressants, Anxiolytics, and/or Hypnotics Users in the General Population

In our sample from the general population, we observed that the prevalence of antidepressant use is 1.9% and <1.0% for anxiolytics and hypnotics (Supplement Table  1).

## 4. Discussion

The aim of this study was to assess the prevalence of antidepressants, anxiolytics, and/or hypnotics use in a large, managed, primary care system of people with T2DM and to determine the sociodemographic characteristics, comorbidities, T2DM medication, and metabolic control associated with its use. We observed that the total prevalence of use was 17.2% in our cohort of people with T2DM. The prevalence of use was 7.1% for antidepressants, 6.5% for anxiolytics and/or hypnotics, and 3.6% for the combination of these medications. In concordance with our expectations, the prevalence of antidepressants, anxiolytics, and/or hypnotics use was higher in people with T2DM, compared to the general population in which less than 2% used antidepressants, anxiolytics, and/or hypnotics [8, 12]. The general population cohort is from the same catchment area as the people with T2DM, but slightly younger. We therefore expect that overall the difference between the general population and people with T2DM would be slightly smaller, but still significant.

In concordance with studies from the literature in the general population, we observed that people with T2DM using antidepressants, anxiolytics, and/or hypnotics were more often female, older, non-Caucasian, and having a lower level of education, compared to nonusers [11, 12, 21]. Several possible reasons have been offered in literature; first women more often suffer from anxiety and depressive disorders, compared to men [22]. Second, women are more likely to use health services for mental problems and thus depression will more often be diagnosed and treated in women [23].

In contrast to earlier research in elderly people with T2DM, we did not observe more comorbidities in users of antidepressants [24, 25]. An explanation for this may be that our cohort was relatively younger and healthier and metabolically well-controlled. We did however observe that anxiolytics and/or hypnotics users had more comorbidities compared to nonusers. This is in accordance with literature in the general population [26].

Furthermore, we were the first to observe with regard to T2DM medication that people with T2DM using antidepressants were more frequently treated with insulin, compared to non-insulin users. This is in accordance with other literatures on the association between depression and T2DM [27] in that it may reflect disease progression. Insulin-treated people with T2DM often have a longer disease history, including poorer glycaemic control and chance on comorbidities [27, 28].

Finally, with regard to metabolic status, no differences in HbA1c level were found between users and nonusers. Earlier studies reported contradictory findings on the effects of antidepressants, anxiolytics, and/or hypnotics use on HbA1c levels [29, 30]. Two longitudinal studies in people with T2DM and one meta-analysis from studies in the general population reported that depression was associated with poorer glycaemic control [29, 30], while one study reported a difference of 6 mmol/mol (0.5%) in HbA1c levels when comparing people with T2DM with and without depressive symptoms [13]. However, another study observed no differences in glycaemic control [14]. Our results support the earlier null-findings; the difference with studies that did find an association may be explained by cohort differences; we are one of the few to have an unselected, population-based cohort of people with T2DM. Finally, longitudinal studies are needed to study the metabolic consequences of these types of medication in people with T2DM.

This study has several limitations that need to be discussed. First of all, we lack information about indication and therefore we decided to combine the anxiolytics and hypnotics into one group. Several anxiolytics, such as oxazepam, can be prescribed in case of anxiety, but also in case of sleep disorders. Further, antidepressants can be prescribed for other disorders than depression alone (e.g., neuropathic pain), but also in cases of anxiety. Also, people may be afflicted by the disease but not have been diagnosed. Finally, we do not have details on psychiatric comorbidities or the onset of the condition with respect to T2DM. Therefore, our current study is not informative on disease prevalence and on the causal effect of medication or the disorder itself on T2DM. We therefore need longitudinal research in large cohorts of people with T2DM, to study the effect of antidepressants, anxiolytics, and hypnotics use on glycaemic control. Another limitation is the absence of data on physical activities and social environment.

The strengths of our study are that we were the first to investigate and report on the prevalence of antidepressants, anxiolytics, and/or hypnotics use, as well as differences in sociodemographic, comorbidities, T2DM medication, and metabolic status between users and nonusers in people with T2DM. In addition, we conducted this study in large (*n* > 5000) population from routine care, which represented all people from a certain catchment area in care for their T2DM, thereby omitting possible selection bias. All information has been accurately registered as part of regular care and therefore provides a unique care-based T2DM cohort.

We conclude that in our managed, primary care system 17% of all people with T2DM used antidepressants, anxiolytics, and/or hypnotics. Users of antidepressants, anxiolytics, and/or hypnotics were more often female, non-Caucasian, lower educated, and more often treated with insulin.

## Supplementary Material

Short description Supplementary material:
The New Hoorn Study is a population-based cohort, which is representative for the general Dutch population. Of the eligible participants, 45% agreed to participate, resulting in a population-based cohort of 2807 participants. As this population-based cohort is sampled from the general population within the catchment area of the Diabetes Care System Cohorts, is about the same age, adheres to the same level of care, and shares a study location, nurses, and study protocols, we believe it is the optimal comparison cohort.
In short, this population-based cohort was aged 53.4 ± 6.7 years; similarly to the T2DM cohort about 50% was male, and, however, as it is a population-based cohort only 7.1% had prevalent diabetes according to the WHO criteria.

## Figures and Tables

**Table 1:** Patients characteristics and prevalence of antidepressants, anxiolytics, and/or hypnotics use in the year 2011 from the Hoorn Diabetes Care System Cohort.

		Percentage missing data
Number of patients (*n*)	7016	
Male (%)	52.2	—
Age (years)	66.3 ± 11.7 (66.6, 15.6)	—
HbA1c (mmol/mol and %)	52 (6.9) ± 1.1 (49, 1.0)	1.8%
BMI (kg/m^2^)	30.2 ± 5.4 (29.3, 6.7)	4.6%
Diabetes duration (years)	7.3 ± 6.6 (6.0, 9.0)	0.3%
Onset age of diabetes (years)	58.1 ± 12.1 (59.0, 16.0)	0.3%
Ethnicity (% Caucasian)	77.5	15
Education (%)		5.4
Low	43.8	
Middle	37.0	
High	13.8	
Marriage status (%)		27.7
Married	45.5	
Unmarried	5.0	
Divorced	0.9	
Single	7.7	
Widowed	13.2	
Only antidepressants use (%)	7.1	—
Only anxiolytics and/or hypnotics use (%)	6.5	—
Combination use (antidepressants and anxiolytics/hypnotics) (%)	3.6	—
Comorbidities (%)	16.5	—
Diet only (%)	17.7	—
Oral glucose lowering agents use only (%)	60.1	—
Insulin use (%)	22.1	—

Data as mean ± standard deviation or median (interquartile range). BMI: body mass index; HbA1c: glycated hemoglobin; SD: standard deviation.

For continuous variables, we reported the mean + SD as well as the median and the IQR.

**Table 2:** Patients characteristics stratified by antidepressant, anxiolytics, and/or hypnotics use in the Hoorn Diabetes Care System Cohort in the year 2011 (*n* = 7016).

	Nonusers	Antidepressants users	Anxiolytics/hypnotics users	Combined use of antidepressants and anxiolytics/hypnotics
*General characteristics*				
Number of patients (*n*) (% of total number of patients)	5806 (82.8)	500 (7.1)	456 (6.5)	254 (3.6)
Male (%)	55.7	38.2^*∗*^	38.2^*∗*^	25.2^*∗*^
Age (years)	65.9 ± 11.5	64.7 ± 13.0	71.7 ± 11.4^*∗*^	68.1 ± 13.2^*∗*^
HbA1c (mmol/mol (%))	52 (6.9) ± 1.1	52 (6.9) ± 1.1	51 (6.8) ± 0.9	52 (6.9) ± 1.0
BMI (kg/m^2^)	30.1 ± 5.4	31.2 ± 5.7^*∗*^	29.9 ± 5.2	31.4 ± 5.4^*∗*^
Diabetes duration (years)	5.9 (2.0–10.5)	5.0 (1.8–10.2)	7.0 (2.8–11.70)^*∗*^	6.0 (2.8–10.6)
*Sociodemographic characteristics*				
Ethnicity (% Caucasian)	78.8	73.8^*∗*^	70.2^*∗*^	70.9^*∗*^
Education (%)				
Low	44.7	51.7^*∗*^	54.7^*∗*^	60.1^*∗*^
Middle	39.9	38.8^*∗*^	34.5^*∗*^	30.0^*∗*^
High	15.5	9.5^*∗*^	10.8^*∗*^	9.9^*∗*^
*Comorbidities*				
Comorbidities (%)	15.4	17.2	26.8^*∗*^	20.1
*Diabetes treatment*				
Diet only (%)	18.2	16.6^*∗*^	14.9	14.2
Oral glucose lowering agents use only (%)	60.3	56.2^*∗*^	62.3	60.2
Insulin use (%)	21.5	27.2^*∗*^	22.8	25.6

BMI: body mass index; HbA1c: glycated hemoglobin; SD: standard deviation.

^*∗*^Significantly different from no antidepressant/anxiolytics/hypnotics users. It is possible that patients are using insulin in combination with oral glucose lowering agents.

**Table 3:** Multivariate regression analyses determining patient characteristics of antidepressants, anxiolytics, and hypnotics use versus no antidepressants, anxiolytics, and hypnotics use.

	Univariate OR (95% CI)	Multivariate OR (95% CI)
Sex (female)	2.29 (2.02–2.61)^*∗*^	2.37 (2.08–2.70)
Age (years)	1.02 (1.01–1.02)^*∗*^	
HbA1c level (mmol/mol)	0.98 (0.92–1.04)	
Comorbidity (myocardial infarction, transient ischaemic attack, stroke)	1.49 (1.28–1.74)^*∗*^	1.64 (1.40–1.93)
BMI (kg/m^2^)	1.02 (1.01–1.03)^*∗*^	
Ethnicity (non-Caucasian)	0.63 (0.55–0.71)	0.65 (0.57–0.74)

Estimated are odds ratios (OR) with 95% confidence interval. *∗* indicates a significant association (*p* < 0.05). The univariate model shows the determinants with *p* values <0.05. The multivariate model shows the final model after backward selection, including variables with *p* values <0.05.

## Additional Points

*Novelty Statement*. (i) Compared to the prevalence in the general population, use of antidepressants, anxiolytics, and/or hypnotics was only slightly higher in people with T2DM. (ii) No differences in comorbidities and HbA1c were observed between users and nonusers of antidepressants, anxiolytics, and/or hypnotics. (iii) Users of antidepressants, anxiolytics, and/or hypnotics were more frequently treated with insulin.
