# Supplementary material for: The Use of Antidepressants, Anxiolytics, and Hypnotics in People with Type 2 Diabetes and Patterns Associated with Use: The Hoorn Diabetes Care System Cohort
Source: Biomed Res Int. 2017 Jan 23;2017:5134602. doi: 10.1155/2017/5134602 (PMC5292378; doi:10.1155/2017/5134602)
Supplement: Supplementary file 1 — Short description Supplementary material: The New Hoorn Study is a population-based cohort, which is representative for the general Dutch population. Of the eligible participants, 45% agreed to participate, resulting in a population-based cohort of 2807 participants. As this population-based cohort is sampled from the general population within the catchment area of the Diabetes Care System Cohorts, is about the same age, adheres to the same level of care, and shares a study location, nurses, and study protocols, we believe it is the optimal comparison cohort. In short, this population-based cohort was aged 53.4 ± 6.7 years; similarly to the T2DM cohort about 50% was male, and, however, as it is a population-based cohort only 7.1% had prevalent diabetes according to the WHO criteria. [file 5134602.f1.pdf]

Supplement

Table S1 Characteristics of general Dutch population stratified by antidepressant/ anxiolytics/ hypnotics use in the year 2007

	**No antidepressant/ anxiolytics/ hypnotics users**	**Only Antidepressants users**	**Only Anxiolytics/ Hypnotics users**	**Combination use of antidepressants AND anxiolytics/ hypnotics**
**General Characteristics**				
Number of patients (n), (% of total number of patients)	2589 (97.1)	51 (1.9)	26 (1.0)	1 (<1.0%)
Male (%)	47.9	25.2*	30.8*	0*
Age (yr)	53.4 ± 6.7	52.0 ± 5.9	52.7 ± 6.4	46.0 ± 0
**Socio-Demographic Characteristics**				
Ethnicity(% Caucasian)	95.9	92.2	92.3	100.0
Education (%)				
Low	25.3	38.0*	44.0*	0*
Middle	47.5	42.0*	40.0*	0*
High	27.2	20.0*	16.0*	100*
**Medication use**				
Metformin use (%)	1.1	0	0	0
Insulin use (%)	0.1	0	0	0

*Significantly different from no antidepressant/ anxiolytics/hypnotics users

Table S2 Patients' characteristics and prevalence of antidepressants and anxiolytics and/or hypnotics use between the year 2007, 2008, 2009, 2010 and 2012.

	**2007**	**2008**	**2009**	**2010**	**2012**
Number of patients (n)	4565	4947	5260	5683	7252
Male (%)	52.0	52.5	52.3	52.7	52.5
Age (yr)	64.9 ± 11.6	65.4 ± 11.6	65.6 ± 11.7	65.6 ± 11.4	66.8 ± 11.7
BMI (kg/m2)	30.2 ± 5.5	30.2 ± 5.5	30.2 ± 5.6	30.2 ± 5.5	30.1 ± 5.4
HbA1c (mmol/mol)	6.8 ± 1.2	6.9 ± 1.2	6.9 ± 1.0	7.0 ± 1.0	6.8 ± 1.1
Diabetes duration (yr)	6.4 ± 6.0<br>(5.0, 6.0)	6.7 ± 6.1<br>(5.5, 7.0)	7.0 ± 6.3<br>(6.0, 8.0)	7.2 ± 6.3<br>(6.0, 8.0)	8.2 ± 6.6<br>(7.0, 9.0)
Education (%)					
Low	39.4	40.4	41.9	44.5	38.7
Middle	30.4	32.4	34.6	37.2	32.6
High	12.2	13.0	13.6	14.2	12.2
Missing	18.0	14.2	9.9	4.2	16.5
Only Antidepressants use (%)	5.4	5.6	6.3	6.8	7.3
Only Anxiolytics and/or hypnotics use (%)	5.8	6.4	6.0	5.8	6.5
Combination use (antidepressants and anxiolytics/ hypnotics) (%)	2.4	2.9	3.1	3.2	3.3
Diabetes treated with Diet only (%)	16.7	15.4	16.5	16.4	18.5
Metformin use, number (%)	60.8	60.7	59.3	59.9	60.0
Insulin use, number (%)	22.5	23.9	24.2	23.7	21.5

Abbreviation: BMI: body mass index; HbA1c: glycated hemoglobin; SD: standard deviation.

For continuous variables we reported the mean + SD as well as the median and the IQR
